# Induced pluripotent stem cells in companion animals: how can we move the field forward?

**DOI:** 10.3389/fvets.2023.1176772

**Published:** 2023-04-25

**Authors:** Laura Barrachina, Tarlan Eslami Arshaghi, Aisling O'Brien, Ana Ivanovska, Frank Barry

**Affiliations:** Regenerative Medicine Institute (REMEDI), Biosciences, University of Galway, Galway, Ireland

**Keywords:** veterinary, regenerative medicine, IPSC, one medicine, horse, dog, cat

## Abstract

Following a one medicine approach, the development of regenerative therapies for human patients leads to innovative treatments for animals, while pre-clinical studies on animals provide knowledge to advance human medicine. Among many different biological products under investigation, stem cells are among the most prominent. Mesenchymal stromal cells (MSCs) are extensively investigated, but they present challenges such as senescence and limited differentiation ability. Embryonic stem cells (ESCs) are pluripotent cells with a virtually unlimited capacity for self-renewal and differentiation, but the use of embryos carries ethical concerns. Induced pluripotent stem cells (iPSCs) can overcome all of these limitations, as they closely resemble ESCs but are derived from adult cells by reprogramming in the laboratory using pluripotency-associated transcription factors. iPSCs hold great potential for applications in therapy, disease modeling, drug screening, and even species preservation strategies. However, iPSC technology is less developed in veterinary species compared to human. This review attempts to address the specific challenges associated with generating and applying iPSCs from companion animals. Firstly, we discuss strategies for the preparation of iPSCs in veterinary species and secondly, we address the potential for different applications of iPSCs in companion animals. Our aim is to provide an overview on the state of the art of iPSCs in companion animals, focusing on equine, canine, and feline species, as well as to identify which aspects need further optimization and, where possible, to provide guidance on future advancements. Following a “step-by-step” approach, we cover the generation of iPSCs in companion animals from the selection of somatic cells and the reprogramming strategies, to the expansion and characterization of iPSCs. Subsequently, we revise the current applications of iPSCs in companion animals, identify the main hurdles, and propose future paths to move the field forward. Transferring the knowledge gained from human iPSCs can increase our understanding in the biology of pluripotent cells in animals, but it is critical to further investigate the differences among species to develop specific approaches for animal iPSCs. This is key for significantly advancing iPSC application in veterinary medicine, which at the same time will also allow gaining pre-clinical knowledge transferable to human medicine.

## 1. Introduction

Veterinary regenerative medicine is a multidisciplinary field with a focus on developing innovative treatments for animal patients. Structural and functional healing of injured tissues and organs can be achieved by using either cells alone, cells combined with tissue engineered constructs or by delivery of the secretome without cells. These strategies involve different therapeutic mechanisms of action with the ultimate objective of enhanced treatment for diseases of veterinary interest (1–5). The advancement of the field is strongly influenced by the One Health—One Medicine approach. By adopting a panoramic view of the challenges, and exploiting the emerging positive outcomes in human and veterinary regenerative medicine, both may advance synergistically. Simply put, valuable insights can be translated in both directions to accelerate translation (6). For example, clinical experience with veterinary species can provide pre-clinical knowledge on safety and efficacy for human application, potentially reducing or eliminating the need for laboratory animals (7, 8). Therefore, a robust and inclusive One Health approach has exceptional value in human and veterinary medicine.

Among different cell types currently being explored for regenerative purposes, mesenchymal stromal cells (MSCs) isolated from multiple species and tissue sources are extensively studied for a wide array of veterinary applications, mostly due to their cell regulatory abilities (9–11). The relative ease in tissue collection, processing and *in vitro* culture has made MSCs an attractive therapeutic option. However, *in vitro* expansion of these cells is limited and extensive passaging may lead to cell senescence. In addition, the differentiation potential of MSCs is limited to mesodermal cell lineages (12–14). Embryonic stem cells (ESCs) can overcome these obstacles as they are pluripotent cells with a virtually unlimited capacity for self-renewal, and can provide a constant source of cells in terms of number and types. However, obtaining ESCs requires the use of embryos, with associated ethical concerns (15). In 2006, Takahashi and Yamanaka reported for the first time an alternative type of stem cell that could overcome the limitations of both adult and embryonic stem cells: the induced pluripotent stem cells (iPSCs). These cells are not naturally occurring but produced in the laboratory: adult somatic cells, such as dermal fibroblasts, can be reprogrammed into pluripotent cells by inducing the expression of four pluripotency-associated transcription factors (Oct4, Sox2, c-Myc, and Klf4, also known as Yamanaka factors). Theoretically, any cells of the body can be transformed into pluripotent cells without the disadvantages associated with ESCs (16). Therefore, the discovery of iPSCs has revolutionized regenerative medicine, not only because of their therapeutic potential but also because of their usefulness for disease modeling, drug screening, and even species preservation strategies (17).

Reprogramming somatic cells into iPSCs involves a global reset of the mature epigenome of the somatic cell, in order to go from its differentiated state back to a pluripotent one. To do that, the endogenous pluripotency-associated genes have to be re-activated while the somatic genes, associated with the specialized function of the cell, need to be repressed. These changes are initiated by inducing the ectopic expression of transcription factors Oct4, Sox2, c-Myc, and Klf4, which are proteins able to interact with the DNA to control gene expression (16, 18). These factors can be delivered to the cell by different methods that may involve or not viral vectors, and may result or not in the integration of transgenes in the genome of the cells (19). Even though the process of delivering the Yamanaka factors may seem relatively simple, complex epigenomic remodeling needs to take place (18). In order to succeed in this process, it is important to consider all the stages of reprogramming, starting from the selection of the somatic cells (20), the choice of the factors and the method to deliver them (19, 21), as well as the signaling pathways that can be regulated by adding growth factors or small molecules to the media (22, 23). Once the cells are reprogrammed, culture conditions need to be optimized to expand iPSCs while maintaining a pluripotent state. Of note, the complexity of the process may result in a mixture of cells at different stages of the reprogramming, thus making it critical a throughout characterization to confirm their identity as iPSCs (24). Therefore, there are many factors that can impact the resulting iPSCs and their subsequent applications. Therapeutic application of iPSCs is a major goal to pursue, however, the complexity of these cells and the genetic changes that they undergo during reprogramming have raised concerns that need to be addressed to move this application forward. Tumorigenicity and immunogenicity are among the main concerns, for which a number of strategies are being developed, from differentiation of iPSCs into specialized cells to the creation of haplobanks (25, 26). While the development of therapeutic applications moves forward, a number of other applications for iPSCs have emerged, being disease modeling one of the most relevant. Generating patient-specific iPSCs allows the subsequent derivation of specialized cells with specific genetic signatures or alterations that otherwise would be extremely complex to obtain primarily from tissues (27). *In vitro* disease modeling can help reducing the need of *in vivo* models and allows involving the species of interest since the pre-clinical phase of drug development. Basic research to unveil mechanisms of disease and physiology, as well as to conduct toxicological studies and drug screening can also be greatly facilitated by iPSCs (28).

Although iPSC technology is still a young field needing intensive work, nonetheless important advancement has been made in the human side (29). However, the veterinary iPSC field is significantly less developed and with much fewer publications. In fact, the first reports on canine iPSCs emerged in 2010 (30), equine in 2011 (31), and felids in 2012 (32). The majority of studies in veterinary species have focused on the generation of iPSCs and/or on their *in vitro* use, mostly to derive cell types relevant for clinical or disease modeling applications, while only very few works have pursued an *in vivo* application. The knowledge on human iPSCs can greatly contribute to advance the veterinary side, as most of the interests and challenges are shared between human and veterinary medicine. However, we also need to increase our understanding on the differences among species (33). Pluripotency networks, epigenomic landscape and identity of the iPSCs, and their derivatives need to be addressed from a comparative perspective rather than directly extrapolating from the human side. Advancing the field of veterinary iPSCs is important to improve the standard point-of-care of companion animals as patients, but also because of their potential as translational models. For instance, dogs suffer several spontaneous diseases with similar pathophysiology and incidence than in humans, like diabetes, epilepsy, or various types of cancers (34). Furthermore, dogs and humans also share the genetic basis of some diseases affecting the cardiovascular, neuromuscular, or immunological systems, thus creating a unique landscape for iPSC-based research (35). The domestic cat can serve as a natural animal model of Alzheimer’s disease (36) or hypertrophic cardiomyopathy (37), and also suffers genetic diseases that affect people too, like retinal blindness or polycystic kidney disease (38). Horses have long been considered as one of the most suitable animal models to study musculoskeletal pathologies like tendon injuries or joint pathologies (39) and, more recently, they are also acknowledged as models for immune-mediated diseases and to study immune responses (40–42). Therefore, joining efforts would revert in mutual benefit for human and veterinary patients, but additionally, there are some animal-specific applications like species conservation for which iPSCs can be of great importance.

**Figure 1:**
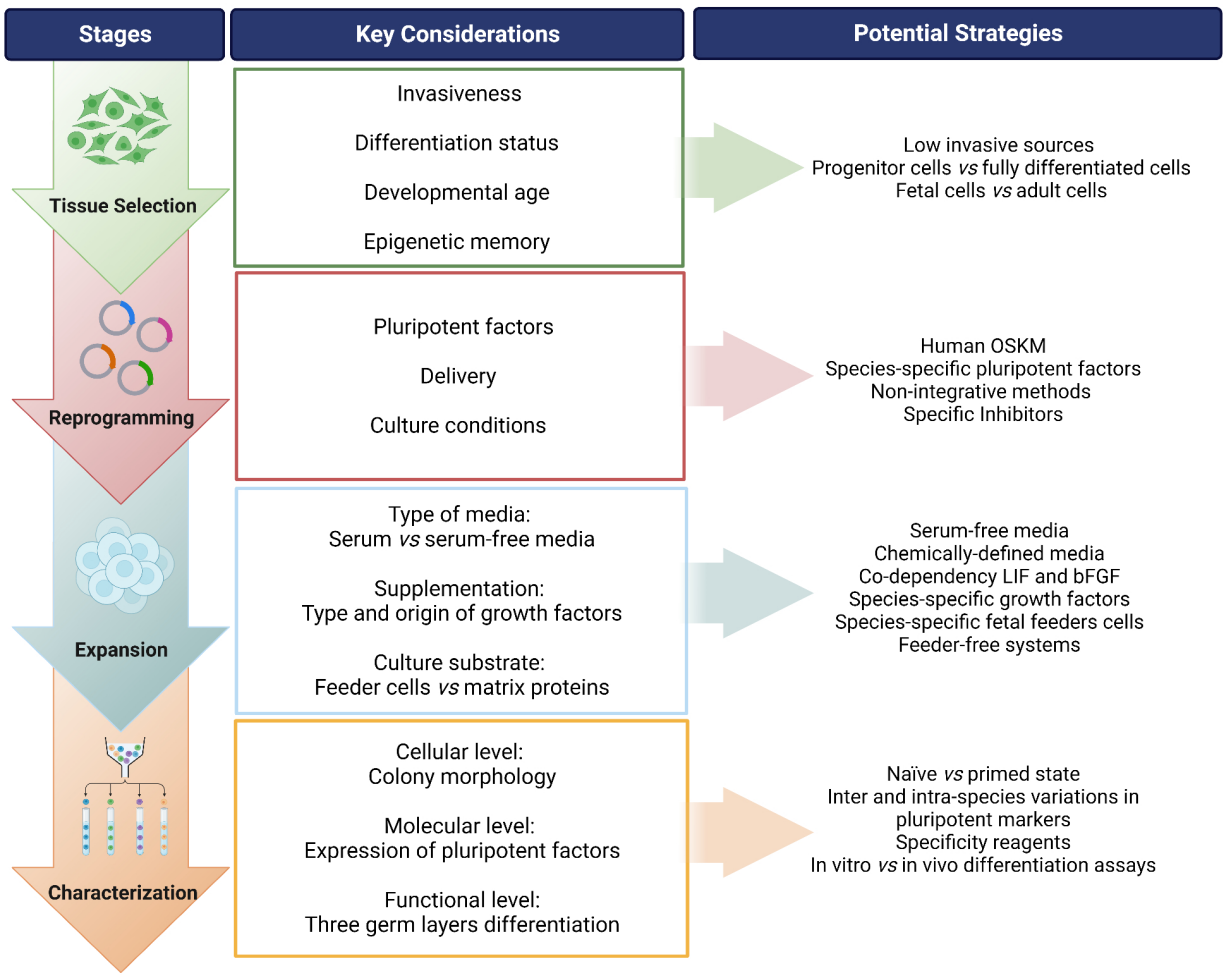
Overview of the process for generating induced pluripotent stem cells (iPSCs) from companion animals. A “step-by-step” flow is presented identifying the stages of the process, along with the key considerations in each step and suggested potential strategies to address each point. OSKM: Oct-4, Sox-2, Klf-4, c-MYC; i.e., Yamanaka factors. LIF, leukemia inhibitory factor; bFGF, basic fibroblast growth factor. Created with Biorender.com.

**Figure 2:**
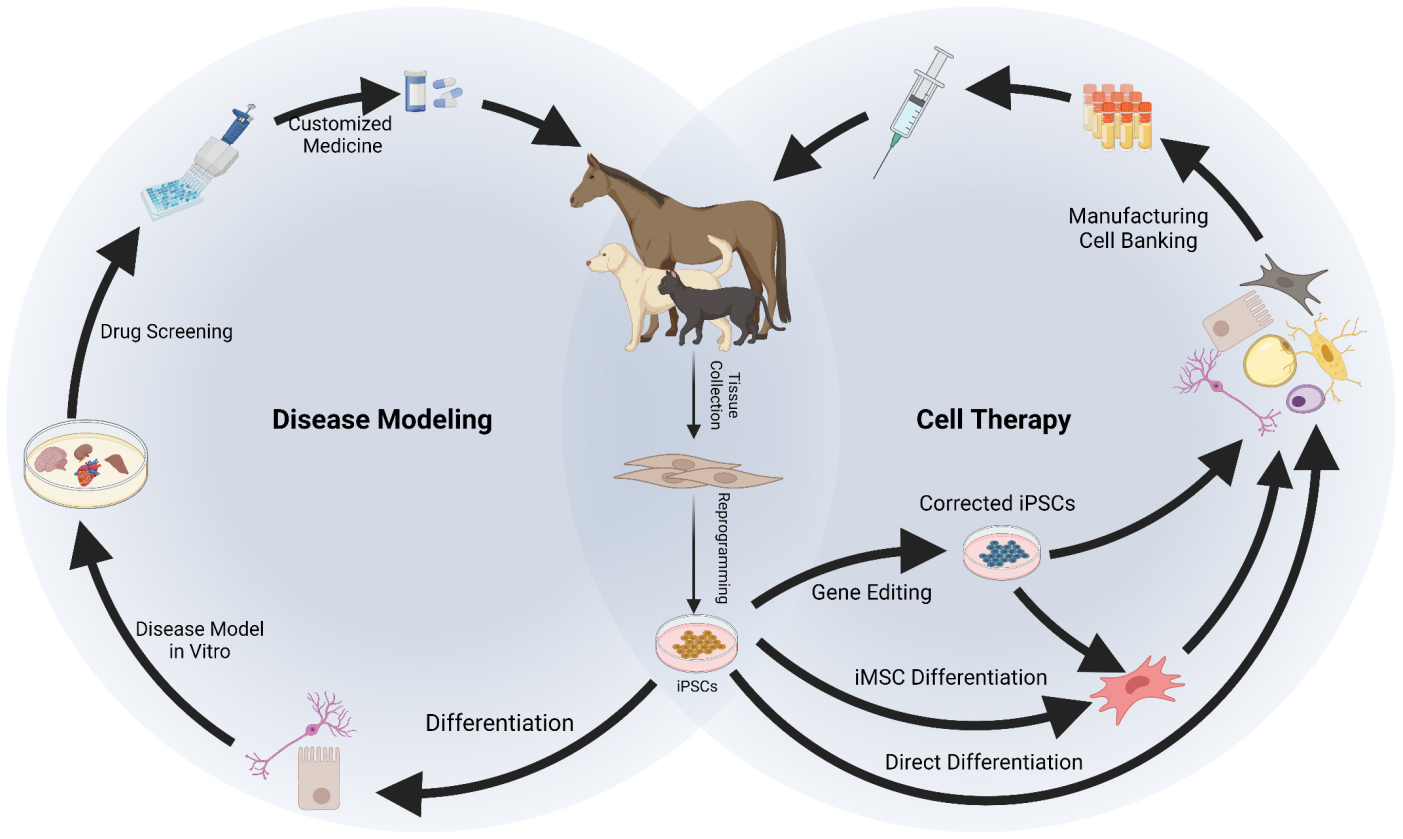
Applications of induced pluripotent stem cells (iPSCs) in companion animals for disease modeling and therapy. After reprogramming, iPSCs can be differentiated into specialized cells to recreate a “disease in a dish.” *In vitro* disease modeling can be used to better understand mechanisms of disease, physiology of the cells and to conduct drug screening, resulting in a more customized approach for therapy. Therapeutic application of iPSCs requires their differentiation into specialized cells or into intermediate progenitors like mesenchymal stromal cells (iMSCs). Gene editing can be used at this stage to obtain healthy cells from patients carrying genetic disorders. The iPSCs, iMSCs, and/or their derivatives can be manufactured and banked for therapeutic use. Created with Biorender.com.

The aim of this review is to provide an assessment of the current state of the art of iPSCs in companion animals, focusing on the equine, canine, and feline species because of their relevance as veterinary patients and their potential as animal models. Following a “step-by-step” approach, we firstly discuss the different stages in the generation of iPSCs from veterinary species (Figure 1) and secondly the development of different applications of iPSCs in companion animals (Figure 2). Our intention is to identify those technical aspects that need further optimization and to provide helpful guidance on future advancements.

## 2. “Step-by-step” approach for generating iPSCs in companion animals

### 2.1. Selecting the tissue source

To obtain iPSCs, the first step is to consider the type of somatic cells to be reprogrammed. Even though theoretically any cell can be induced into a pluripotent state, there is evidence that some cells are more easily reprogrammed than others. In addition, the invasiveness of the cell harvest procedure and the ease of culture should be taken in to account. Human iPSCs have been established from a wide range of tissue sources including dermal fibroblasts (43), peripheral blood mononuclear cells (PBMCs) (44), bone marrow derived MSCs (BM-MSCs) (45), and adipose derived stromal cells (ADSCs) (46). PBMCs are easily accessible while the other cell types require more invasive intervention. Indeed, waste or discarded tissues, such as foreskin fibroblasts (47), periodontal tissue (48), or renal cells from urine (49) have also been taken used. Umbilical cord blood banks are also a useful resource for iPSC reprogramming (50, 51).

The differentiation status of the cell can influence the reprogramming efficiency, as the epigenomic state needs to be reset to a pluripotent state. For instance, hematopoietic stem and progenitor cells can be reprogrammed into iPSCs much more efficiently than terminally differentiated lymphocytes (52). In addition, the developmental age of the cells can affect their capacity to revert to an earlier state of pluripotency, as shown by the fact that embryonic and fetal tissues may be reprogrammed more efficiently than adult tissues (53). Furthermore, iPSCs are reported to retain epigenetic memory of the parent cell type (54, 55), which may influence the differentiation potential of iPSCs toward a desired lineage. For example, iPSCs derived from human cardiac-derived mesenchymal progenitor cells and pancreatic islet beta cells demonstrated enhanced differentiation toward the parent lineages compared to cells from other tissues (56, 57).

In companion animals, adult and fetal fibroblasts have been the most commonly used to obtain iPSCs, but other cell types have been explored such as keratinocytes (58), MSCs from different tissue sources (adipose tissue, bone marrow, umbilical cord tissue, and peripheral blood) (59–61), myogenic mesoangioblasts (MAB) (60), tenocytes (62, 63), and PBMCs (64). Very few studies have directly compared the generation of iPSCs from different cell types in these species.

In horses, Pessôa et al. (59) reported that the tissue of origin of the cell may significantly influence the capacity for reprogramming. Adult fibroblasts, umbilical cord tissue (UC)-MSCs, and adipose tissue (AT)-MSCs were successfully reprogrammed, with AT-MSCs showing the highest colony formation potential, whereas BM-MSCs did not produce iPSCs. In the same study, authors observed differential miRNA expression profile among iPSC lines, which may be the result of different responses to reprogramming. Direct comparison of iPSC generation from cells of adult and fetal origin has not been performed in horses. However, when comparing fibroblasts from young and old individuals, it was suggested that the derivation of equine iPSCs is not impaired by aging (65).

Regarding canine iPSCs, it seems that adult cells are more “resistant” to reprogramming compared to fetal cells. Questa et al. (66) hypothesized that chromatin remodeling and accessibility were behind this resistance to reprogram since chromatin remodeling is required for the inactivation of somatic loci and activation of pluripotent ones. Therefore, these authors explored the implications of chromatin accessibility for canine somatic cell reprogramming by comparing different embryonic and adult cell types. The transduction efficiency was similar between adult and embryonic cells, however, only iPSCs from embryonic origin met pluripotent criteria whereas adult reprogrammed cells did not form stable colonies. Authors identified global patterns of chromatin openness, finding that iPSCs and embryonic fibroblasts shared substantially more features than iPSCs with adult cells. Actually, adult canine cells showed a region of closed chromatin that was open in embryonic cells and in which pluripotency associated genes are located. Findings were aligned with that expected during reprogramming and may explain why adult cells are more ‘resistant’ to reprogramming, which may help enhancing the process by targeting reprogramming barriers.

The influence of epigenetic memory in the differentiation potential of iPSCs has not been deeply studied in companion animals, but it has been suggested that equine iPSCs retain some lineage commitment since those originated from MAB formed a higher quantity of muscle patches in teratomas, while iPSCs from PB-MSCs produced larger chondrogenic patches (60). The same group similarly showed that canine iPSCs derived from MAB had enhanced propensity to differentiate into skeletal muscle lineage compared to iPSCs derived from fibroblasts, which could be attributed to the DNA methylation pattern of MAB (67). Furthermore, authors also differentiated canine iPSCs from MAB and from fibroblasts into mesodermal progenitors (MiPS) and administered them in dystrophic mice, showing that the engraftment in the skeletal muscle was higher when MAB-MiPS were delivered compared to fibroblast-MiPS.

Evidence in companion animals is still limited to suggest superior cell sources for iPSC reprogramming, but collectively with human evidence points at carefully considering this choice. Not all cell sources are equally suitable for reprogramming and iPSC properties can be impacted by their origin, so identifying the most suitable source for a particular application is of utmost importance. To do that, it is also important to unveil and understand the differences among distinct cell types in each species, particularly at the epigenomic level.

### 2.2. Reprogramming

#### 2.2.1. Inducing the expression of pluripotent factors

Once the tissue source is selected, the choice of the pluripotent transcription factors and the method to deliver them to the cells should be carefully considered. Studies on companion animal iPSCs have mostly used human or murine factors for reprogramming, typically the four Yamanaka factors (OSKM) (16, 43). Of course, the mRNA and protein sequence homology of these transcription factors should be as high as possible. For example, in the horse, the homology is higher with human sequences than with mice (68) and, even though some works have generated equine iPSCs using murine factors (31, 69, 70), the studies that have directly compared both of them reported success only with human factors (59, 68). Studies comparing human and murine factors to generate canine iPSCs found that both were able to reprogram canine cells, but only human transgenes were silenced (71). To the best of our knowledge, only one early canine iPSC study was carried out using species-specific factors (30). Later studies used factors of human or murine origin. Based on what we understand to date, it is unclear whether species-specificity of reprograming factors is important for iPSC generation in companion animals (72). Interestingly, while the four OSKM factors are sufficient to reprogram equine and canine cells, it seems that the addition of NANOG is key in felids (32), including the domestic cat (73). Based on these considerations, it seems especially prudent to pay attention to the species origin of the factors, as well as the specific combination of factors used.

Methods used to induce expression of the selected pluripotent factors may be classified as integrative/non-integrative and viral/non-viral. Human and murine iPSCs were originally generated using integrating retrovirus and lentivirus vectors to introduce the OSKM reprogramming factors (16, 43, 74). Genome-integrating methods may result in heterogeneous iPSC lines that are not suitable for clinical applications because the transgenes may become reactivated in iPSC derived cells, leading to a risk of tumor formation (21, 75). This is a limitation that will be further discussed in the Application section. Transgene free, non-integrating methods have been utilized to overcome these safety concerns, including Sendai-virus (76), episomal vectors (77, 78), and RNA based methods (79, 80). Furthermore, clinical-grade human iPSCs have been developed using modified mRNAs and non-integrating episomal vectors (45, 81). However, while significant advancement has been accomplished in the generation of human iPSCs by non-integrative methods, these strategies have been rarely applied in companion animals, with the majority of applications involving integrative viral methods (35, 82).

In the generation of equine iPSCs, retroviral vectors were mainly used in early efforts and lentiviral vectors more recently. Specifically, the use of a STEMCCA cassette can increase the efficiency of reprogramming by delivering the four factors together and thus also reducing the number of integrations in the genome (83). Only a few studies in equine iPSCs report the use of non-viral methods. The Piggy-back transposon technique was indeed used in the first report on equine iPSCs (31), and the lines obtained were also used in later studies (84, 85). However, Moro et al. (86) compared the lentiviral and transposon systems and found that only the former was efficient at generating equine iPSCs from fetal fibroblasts. Transposon reprogramming allows more control of transgene expression by using excisable or inducible systems, but it is still an integrative method. Transgenes can also be excised if delivered by lentivirus, as done for example by Chauveau et al. to generate canine iPSCs (87). A relatively simpler way of controlling the expression of transgenes is by using an inducible promoter, however the transgene remains integrated (31, 70, 88). In general terms, these strategies can improve the safety profile of integrative methodologies, but integration still takes place with the potential risk associated with activation of unwanted genes such as those related to tumorigenicity.

Exploration of transgene-free strategies has been more extensive in canine iPSCs, however direct extrapolation of the conditions used for human iPSCs does not seem to be straightforward. Baird et al. (61) used both retroviral and Sendai-virus based delivery in the same cells, but iPSC colonies appeared only with the former (61). Chow et al. also used the Sendai-virus system and reported that only a single colony was viable upon further passaging after colony picking (89). However, this was sufficient to establish a line that was used in this and subsequent studies (90). Similarly, Tobias et al. could not maintain stable canine iPSCs for longer than 26–30 days after reprogramming when using Sendai-virus (91). Tsukamoto et al. generated canine iPSCs with Sendai-virus that could be maintained for multiple passages, but only one line was obtained which failed to produce all three germ layers in teratoma assays in mice, suggesting that it might be a heterogeneous population (64). Later efforts by the same group showed that canine iPSC generation with Sendai-virus is possible but requires very specific conditions, including supplementation with a cocktail of small molecules. With this improved protocol, these authors were able to culture the generated canine iPSCs over 40 passages (92). Other non-integrative virus strategies have been tested for canine iPSC generation, such as the use of a vector based on the Venezuelan equine encephalitis RNA virus, which overall was not successful (93).

Non-viral and non-integrative methods for pluripotent factors delivery have also been explored for canine iPSCs. Yoshimatsu et al. (94) reported on the use of episomal vectors delivered by electroporation. While colonies could be obtained with this method it was only when highly defined media was used, and still the reprogramming efficiency was very low (94). Chandrasekaran et al. also used episomal reprogramming with electroporation, but even though morphological changes were observed in the cells, complete reprograming was not achieved and lentivirus was used subsequently, resulting in generation of canine iPSCs from the same somatic cells (95).

Although iPSCs generated with integrative methods can be effectively used for *in vitro* applications (basic research, disease modeling, drug screening, etc.) (96), the optimization of non-integrative methods is needed in the veterinary field to develop safer therapeutic applications. In spite of the efforts of several groups, we currently do not have a robust and widely used tool for transgene-free obtainment of iPSCs in companion animals. One possible reason may be that in companion animals iPSCs, the continuous expression of the exogenous transgenes may be required to maintain the pluripotency, as the endogenous networks might not be fully activated (33) thus significantly dampening the derivation of stable transgene-free lines. Therefore it is key to better understand such pluripotency networks in animals to provide the required conditions for iPSC generation.

#### 2.2.2. Culture conditions during reprogramming

Following delivery of the pluripotent factors, appropriate culture conditions are needed to facilitate the changes in gene expression that allow the cell to alter its fate from somatic to pluripotent. This process can be facilitated by inhibitors of certain protein kinases, like glycogen synthase kinase 3 (GSK3), mitogen-activated protein kinase (MAPK), MAPK/extracellular signal-regulated kinase (MEK), Rho-associated kinase (ROCK), or activin-like kinase (22). Furthermore, histone acetylation facilitates the binding of transcription factors to DNA, so chemical inhibitors of histone deacetylase (HDAC) such as valproic acid, sodium butyrate, or ascorbic acid can increase chromatin accessibility and thus potentially improve reprogramming efficiency (23).

Only a few equine iPSC studies have reported the use of kinase inhibitors (31, 97) or HDAC inhibitors (65) during reprogramming. However, these studies have not compared different combinations and neither have explored in detail the specific changes elicited by the inhibitors. In feline iPSCs, in spite of the limited number of studies published, the use of inhibitors has been reported (98). Optimization of iPSC generation has been further pursued in dogs. For instance, Moshref et al. (99) hypothesized that HDAC inhibitors would increase chromatin accessibility and facilitate reprogramming of adult canine cells. These authors found that neither valproic acid nor sodium butyrate effectively inhibited canine HDAC. On the other hand, panobinostat, another HDAC inhibitor, significantly increased histone acetylation and improved chromatin accessibility but without evidence of increased efficiency of generating iPSCs (99). Furthermore, Kimura et al. found that a cocktail of small molecules including some of the inhibitors mentioned (ROCK inhibitor, MEK inhibitor, GSK3b inhibitor, TGFβ antagonist, forskolin, and ascorbic acid) contributed to efficient generation of canine iPSCs (92).

These findings certainly appear to indicate that each species has a unique epigenomic landscape that requires specific approaches, and this might help to explain the relatively limited success in obtaining iPSCs in companion animals. While human studies can provide a basis of knowledge, directly extrapolating the same protocols into other species would not be an optimal strategy. Thus, more studies in this direction are needed to understand the conditions required for reprogramming in each species.

### 2.3. Expansion of iPSCs in companion animals

Once the cells are reprogrammed, the next stage is to maintain them in a pluripotent state and this generally requires the use of specific media containing selected growth factors and chemical components, as well as layers of feeder cells or matrix proteins (100, 101). Media composition for expansion of veterinary iPSCs has been reviewed elsewhere (35, 82), so this review will only focus on two aspects directly related to species-specific aspects and transferability.

One of these is the use of either serum-containing or serum-free media, the latter being more suitable for therapeutic applications as it avoids potential xeno-contaminants and/or infectious diseases, as well as reduces batch-to-batch variation. Nevertheless, it should be noted that some serum-free media may still contain components of human or animal origin that are potential xeno-contaminants for veterinary species. Furthermore, it has been shown for animal MSCs that serum-free media developed for human cells may not work as well in veterinary species [reviewed by (102)]. For iPSCs, it seems that serum-free conditions may work better in equine species. Some papers have reported success in using fetal bovine serum (FBS), but studies directly comparing FBS vs. knockout serum replacement (KOSR) reported better results with the latter (69). In canine iPSCs, a majority of works have also used serum-free media [reviewed by (35)], however other studies suggest that media containing FBS result in higher colony formation compared to KOSR during reprogramming (91). On the other hand, reports in felid iPSC suggest that FBS-containing media are more advantageous (32, 73, 98, 103).

A second important aspect for veterinary iPSC media composition relates to growth factor requirements. The dependence of iPSCs on either basic fibroblast growth factor (bFGF) or leukemia inhibitory factor (LIF) is related to the stage of the embryonic development that is mimicked by pluripotency induction. ESCs from the inner cell mass present a more naïve phenotype, with mounded colonies that are dependent on LIF. When ESCs are derived from the epiblast, a structure formed later during the development, these cells seem to be already primed, possibly representing a more restricted state of pluripotency, and are dependent on bFGF with colonies presenting a flat morphology (33). The majority of human iPSC lines resemble the primed phenotype (104), however there are mixed reports on the naïve/primed nature of iPSCs from companion animals (24). The scarce literature in felid iPSCs points at LIF-dependency (32, 73, 98, 103) while only bFGF-dependent (59, 60, 68, 71) and only LIF-dependent (70, 97) iPSC lines have been reported in both equine and canine species; however, the evidence so far points at a co-dependency on both factors in these two species (69, 105, 106). The reason for this co-dependency is not well understood. A study in canine iPSCs found that bFGF would act by inhibiting spontaneous differentiation toward ectoderm and mesoderm, while LIF activated the JAK-STAT3 pathway involved in pluripotency maintenance, but in a different manner than described in mouse ESCs (107). These studies collectively show that iPSCs from different species may present unique mechanisms for maintenance of pluripotency. Understanding such mechanisms is critical to provide the optimal conditions for expansion of iPSCs for different applications in the veterinary field. Moreover, LIF and bFGF used in animal studies are usually from human or murine origin. The use of species-specific factors has been suggested (108) but scarcely reported. Interestingly, only feline LIF, but not murine LIF, can maintain the pluripotent features of iPSCs in the domestic cat (73). Species-specific reagents usually present more limited availability (102), but may represent an important strategy to enhance pluripotency maintenance in these species.

In addition to specific media composition, iPSCs require to grow onto layers of feeder cells, for which inactivated mouse embryonic fibroblasts (iMEF) are commonly used. This possess another concern when the application of interest is therapeutic: the presence of xeno-contaminants in the cell products, or even the risk of disease-transmission. A possible strategy to avoid xeno-contamination at this point is the use of feeder cells from the same species. For example, human iPSCs can be cultured onto neonatal foreskin fibroblasts with good results (109). Similarly, Nagy et al. used 1:1 iMEF and equine fetal fibroblasts (31), and Zhou et al. used cat fetal fibroblasts (98) to prepare feeder layers. As discussed above, fetal cells can be more easily reprogrammed, and the same cells could be used as feeders after inactivation. This strategy would be more time-consuming and less standardized than the purchase of batch-tested, ready-to-use iMEF, but it might be interesting to explore whether using species-specific feeder cells could better support iPSCs, in addition to avoid xeno-contamination.

For the development of clinical grade hiPSCs, standardized and quality-controlled xeno-free and feeder-free culture products are commercially available including media such as Essential E8 (Gibco), mTeSR plus (Stemcell Technologies), StemFit (Ajinomoto) and NutriStem (Sartorius) (110–113), and matrix substrates that are more defined with less batch-to-batch variability, such as vitronectin (114), laminin-521 (115) or-511 (116), CellStart (117), and synthetic materials (118). The use of such systems is much more rapidly evolving for human iPSCs (100, 101), while most reports in companion animals rely on feeder cells and mostly on iMEF (24, 35, 82). Some attempts have been done to adapt the use of commercially available iPSC media and feeder-free substrates formulated for human iPSCs into companion animal cells. Such systems present the advantage of having a defined composition, being more stable and homogeneous, and providing serum-free, cell-free, or even xeno-free conditions. In horses, there are only brief mentions to the use of the StemFlex system (Thermofisher) to maintain equine iPSCs once the lines were established (62, 63). In cats, StemFlex media was used for reprogramming and expansion of feline iPSCs but supplementation with LIF and protein kinase inhibitors was needed (98). In dogs, Kimura et al. compared the suitability of different commercial media and feeder-free substrates and found that StemFit media (Ajinomoto) and iMatrix-511 (Nippi) provided the most suitable conditions for canine iPSC maintenance and large-scale expansion, and even LIF could be removed (108).

In summary, similarly to that discussed for previous steps, there are not standardized culture systems for iPSCs from companion animals, being of great importance to develop serum-free and feeder-free options for clinical application. This would require fine-tuning of the conditions and a more in-depth understanding of animal iPSC requirements, along with increasing availability of species-specific reagents to truly avoid xeno-contamination.

### 2.4. iPSC characterization in companion animals

Once iPSC putative lines are established, it is critical to confirm that these really are pluripotent cells. iPSCs are characterized at three levels: cellular (morphologically), molecular and functional. The cells should have a large nucleus and form compact colonies. They should endogenously express pluripotency markers at both gene and protein levels, and have the potential to spontaneously differentiate into the three embryonic germ layers [either *in vitro* via embryoid body (EB) formation assays, and/or *in vivo* via teratoma assay in immunocompromised mice]. Finally, iPSCs must have a stable karyotype as they can acquire chromosomal abnormalities after genetic reprogramming and long-term culture (43, 119, 120). Reports on the characteristics of iPSCs from companion animals at these three levels have been reviewed and compared by other authors (24, 35, 82). A detailed description is out of the scope of this review, but instead we are highlighting the main aspects to consider at each level of characterization.

First, and as discussed in the previous section, at the cellular level it is not clear which type of colony morphology features each species of companion animals. Both naïve and primed-like morphology have been described, which also relates to the dependency of these cells on different growth factors. Thus, so far we do not have a strict criteria at this level to consider the cells as iPSCs in each species. This also adds complexity to the selection of colonies when these start emerging from reprogrammed cells. Colonies are picked individually mostly based on their morphology and ideally should be monoclonal, i.e., starting from a single reprogrammed cell. This process is challenging and labor intensive, and can lead to a heterogeneous selection of lines that are apparently similar but hold subtle phenotypic differences. Such differences may be very difficult to appreciate at the morphological level but could eventually result in different characterization profiles and varying differentiation capacity (58).

Second, at the molecular level, different works have reported the expression at the gene and/or protein level of several pluripotent factors. It is important to note two points in this regard: the lack of a standardized panel of markers, and the relevance of ensuring the specificity of antibodies and primers used for characterization. Human and murine pluripotent cells have shown differential expression of certain markers (121), and this variability is also expected across veterinary species. To determine which pluripotent markers are expected in each species we could look at the expression pattern in ESCs. However, intra-species variability has also been noted for ESCs. For instance, in horses and dogs, the same marker has been reported as both positive and negative in ESCs of the same species [reviewed by (24)]. Furthermore, the limited number of ESC lines derived from companion animals makes it difficult to elucidate whether ESCs and iPSCs are truly equivalent and what developmental stage reflect in these species (24). An additional obstacle for the analysis of pluripotent markers in veterinary species is the complexity of finding antibodies which are either species-specific or presenting good cross-reactivity with the species of interest. Whereas the availability of suitable antibodies for veterinary species has substantially improved in the last years, it still can be difficult to find reliable antibodies for some specific markers. Moreover, the use of different clones for the same marker among different studies might contribute to the heterogeneity observed intra-species (24). The analysis of gene expression is also an important tool and designing primers specific for the species of interest is easier than developing antibodies. However, because of the high homology in the mRNA sequences between the human exogenous factors used for reprogramming and the endogenous genes activated in the cell (68), it is critical to ensure that the primers are only amplifying the target of interest.

Finally, variable outcomes have been reported in the different species when it comes to the functional pluripotency of iPSCs, i.e., their ability to differentiate into cells of the three germ layers. The *in vitro* EB formation assay has provided more consistent results, which have shown successful differentiation of iPSCs. However, *in vivo* formation of teratomas has not been observed in all the reports in companion animals, or only partial differentiation has been recorded (24, 82). A potential explanation for this would be an incomplete reprogramming of the cells into the pluripotent state, even though the other criteria are met.

As aforementioned, different studies have used different methodologies and conditions for reprogramming and culture, and this lack of standardization could probably influence differences observed in the characterization of iPSCs in companion animals (24). Thus, it is imperative to advance in determining the pluripotent features representative of each species and in developing suitable methods to analyze them with confidence. Standardization of iPSC characterization in companion animals is key to develop robust applications, and is tightly influenced by a previous proper establishment of reprogramming strategies and maintenance conditions.

## 3. Applications of iPSCs in companion animals

The use of iPSCs finds multiple applications allowing development of novel treatments in human and veterinary medicine. These applications range through various biomedical disciplines, including development of cell therapies, disease modeling and drug testing, and clinical application for untreatable diseases in both people and companion animals (35, 122, 123). In addition, as a characteristic application in animal species and important for the maintenance of biodiversity, iPSCs have been generated from critically endangered mammalian (32, 103, 124, 125) and avian species (126). These are important for the study of developmental and physiological species-specific processes, and as wildlife preservation efforts they are important for the conservation of genetic resources and maintenance of a healthy eco-system.

When it comes to the applicability of iPSCs in companion animals, reports are scarcer than for their generation and mainly focus on therapeutics or disease modeling. Therapeutic use of iPSCs has been mainly proposed for musculoskeletal conditions in horses (60–63, 88, 127–129) and for neurological and cardiovascular conditions in dogs (67, 95, 130–133). In terms of disease modeling, equine iPSCs can also be used for neurological conditions (58, 134). In dogs and humans, there is an interest in modeling genetic diseases by deriving patient-specific iPSCs with genetic abnormalities, which poses a remarkable scenario for translational medicine (35). In cats, to the best of our knowledge, there are no reports on therapeutic or disease modeling approaches, being the bibliography in this species the most limited. However, interestingly, iPSCs have been proposed in wild feline species as strategy to preserve biodiversity (32, 103).

The different applications of veterinary iPSCs clearly remain less developed than in human research, but hold a similarly high potential yet to be explored. Nevertheless, the challenges to accomplish safe therapeutic application are essentially the same in all species. Some of these limitations arise during the generation process, as has been exposed in the first part of this review, or are inherent to the nature of these cells. In this section, we will review the current state of the art of iPSC applications in companion animals and their main limitations (the present), and we will discuss potential strategies to address such challenges and to keep moving the field forward (the future).

### 3.1. Where are we: present of iPSC applications in companion animals

#### 3.1.1. iPSC-based cell therapy in companion animals

The current clinical investigation of iPSC-derived cell therapies in companion animals is still limited as the field is in its initial stage. The rationale behind the interest on these cells is that they could act as direct replacement of diseased cells with healthy and functional cells able to re-establish the tissue homeostasis, which results in an approach closer to actual tissue regeneration than with adult stem cells. The published studies in veterinary species so far offer insights on proof of concepts and initial clinical evidence in low number of clinical cases. In equines, based on the interest in treating recurrent sport injuries, the majority of studies focused on generating iPSC-derived cell types clinically relevant for musculoskeletal and wound healing injuries. In this regard, equine iPSCs have been differentiated into several cell types including osteoblasts (129), chondrocytes (60), tenocytes (62, 63, 127), myocytes (128), and keratinocytes (85). In spite of the *in vitro* evidence of iPSC differentiation potential, the functionality of the obtained cells has only been demonstrated to certain extents and would need to be further tested in the clinical setting. Similarly, the transplantation of functional neuronal cells derived from iPSCs would be of great benefit in the specific context of the nervous system, characterized by very limited regenerative abilities and accompanied by the incidence of neuropathies and traumatic spinal cord injuries in canine and equine patients. Based on this, equine functional motor neurons have been generated (58) and canine iPSC-derived neuronal progenitors have been tested for the treatment of traumatic spinal cord injury in three canine patients. While adverse effects were not noted, neither clinical improvement nor tissue remodeling were observed up to a 1-year follow-up (131).

Thus, besides *in vitro* evidence of iPSC differentiation, optimal clinical use of iPSC-derived cells requires demonstration of cell engraftment and functionality *in vivo*. A proof of concept study using autologous canine iPSCs showed successful myocardial delivery in healthy dogs as monitored by non-invasive imaging techniques. Additionally, these canine iPSCs were differentiated into endothelial cells and administered *in vivo* into two murine models: hind limb ischemia and myocardial injury, in both of which these cells suggested efficient functionality (130). Similarly, canine iPSC-derived mesodermal progenitors showed engraftment and functional improvement in a murine model of cardiac and skeletal muscle, with no off-target tissue formation (67). A murine model of muscle injury was also used to test equine iPSC-derived myofibers, which showed engraftment and histological improvement, but the regeneration was not complete (70).

An alternative option for delivering therapeutic cells is by generating MSCs from iPSCs (iMSCs) as an intermediate cell type that can be further used for cell therapy development with a major interest in musculoskeletal applications in companion animals. Canine iMSCs have been successfully differentiated into osteogenic and chondrogeneic lineage (90, 135). Furthermore, from a functional perspective, canine iMSCs show an immunomodulatory capacity similar to primary MSCs derived from adipose tissue and bone marrow, with similar gene expression profiles, effects on the proliferation of T cells, maturation of dendritic cells and response to priming with pro-inflammatory cytokines (89, 136). Furthermore, canine iMSCs IV injected in three healthy dogs did not produce adverse events in the short term, neither tumor formation was observed up to 15 months of monitoring (89). Equine iMSCs have also been tested *in vivo* for the treatment of a variety of naturally-occurring musculoskeletal injuries in equine patients, showing overall positive effects with absence of serious side effects (88). The heterogeneity of conditions included in this study and the lack of a control group hamper extracting definitive conclusions, but the results are valuable as a proof of concept for the therapeutic potential of iMSCs in horses.

#### 3.1.2. iPSC-based disease modeling and drug screening in companion animals

Efficient prevention and treatment of diseases requires advanced knowledge of the altered genes and pathways responsible for the diseased phenotype of interest. To better understand the molecular background and establish the appropriate treatment, iPSCs can be exploited as *in vitro* models of disease. Furthermore, the defect of interest can be induced and subsequently multiple compounds of interest can be tested to identify a candidate drug with higher therapeutic efficiency (24, 27). In addition, when it comes to genetic disorders, genome editing tools like CRISPR-Cas9 technology can be used to correct the mutation and generate isogenic iPSC lines as controls. This allows accounting for the influence of the genetic background, since the isogenic line only differs from the original one in the disease-causing mutation (137).

Compared to current advances in the use of human iPSCs in disease modeling and drug screening, there is scarce published literature in companion animals and is mostly limited to neurological disorders. In canines, an iPSCs line was generated from a West Highland White Terrier affected by mild cognitive impairment, showing an important proof of concept on successfully generating iPSCs from a geriatric patient (95), while on the equine side iPSC-based *in vitro* models have been generated to study the process of neurotropic viral infections (134). Although limited, these studies constitute relevant milestones for generating efficient *in vitro* modeling systems that will not depend on limitations of adult somatic cells and can eventually lead to a personalized/customized medicine approach where the most efficient treatment will be made available in a patient-centered approach.

#### 3.1.3. Limitations for iPSC applications in companion animals

The iPSC field comes with as many promises as challenges, the latter being even more present in veterinary medicine. Potential applications of iPSCs are almost endless for therapy and research, however unleashing all of this potential requires overcoming several limitations, owed to the complexity and particularities of these cells. The process of generating iPSCs in companion animals faces several challenges itself, as detailed in the first part of this review. Furthermore, once iPSCs are generated, their posterior use for *in vitro* or *in vivo* applications does not come without limitations. Some of these limitations directly arise from the generation stage, such as transgene expression or xenogeneic contamination already discussed, while other handicaps derive from the inherent characteristics of these cells.

One of the key challenges, particularly for *in vivo* application, is the pluripotent nature of these cells. As aforementioned as part of the functional iPSC characterization, these cells have the potential to form benign tumors composed of multiple cell types, known as teratomas, if they are administered undifferentiated in immune-compromised recipients (138). This can pose significant health risks for the recipient and limit the use of iPSCs in medical or veterinary applications. Because of this, and as it will be discussed later, *in vivo* applications aim at using differentiated cells derived from iPSCs that have lost their pluripotency. Furthermore, iPSCs also possess a risk for malignant tumorigenesis. This risk is particularly concerning if integrative methods are used for reprogramming, which are so far the most commonly reported in veterinary species. The random integration of the transgenes into the genome of the cell can activate tumorigenic genes and, even if transgene expression is silenced, they are still present and can reactivate even after differentiation. This is particularly concerning for *in vivo* applications, but can also impact the outcome of *in vitro* research and applications if iPSCs become tumorigenic (25).

Another consideration concerning the therapeutic use of the iPSCs is their immunogenicity. As it will be discussed later in the Banking section, the use of allogeneic cells presents several advantages, particularly in the case of iPSCs which obtainment is highly demanding. However, the immune system of the recipient may recognize and target allogeneic cells, thus affecting the effectiveness and safety of the therapy (139). Importantly, even if the iPSCs are autologous, i.e., derived from the own patient, they can still be rejected by the immune system. This autologous rejection may be related to different factors. iPSC-derived cells are often immature and thus express low levels of the major histocompatibility complex (MHC), which make them targets of natural killer cells. In addition, the genomic and epigenomic changes that the cell undergoes during reprogramming and subsequent *in vitro* expansion and differentiation may result in immunogenic triggers (26).

Another hurdle for the therapeutic application of iPSC derivatives is the lack of proper function, where differentiated iPSCs may not function properly *in vivo*, especially if they are not fully matured or are not adequately integrated into the recipient tissue (140). This issue will be covered in the next section along with strategies to enhance applicability of iPSCs in companion animals. Finally, the use of iPSCs in medical applications is also highly regulated and requires regulatory approval, which can significantly slow the development and commercialization of iPSC-based therapies. Furthermore, the development and production of iPSCs is still a relatively new, complex and expensive field, limiting the accessibility of iPSC-based therapies for many patients (96, 141, 142).

### 3.2. Where are we going: future of iPSC applications in companion animals

#### 3.2.1. Differentiation of iPSCs into specific cell types

Most iPSC applications require their differentiation into the desired cell type, either if they are used for therapy, for disease modeling or for drug screening. Differentiating the iPSCs substantially reduces the potential risks associated with teratoma formation and facilitates the regulatory approval process. Additionally, these differentiated cells are more mature and functional, which can increase their effectiveness and reduce their immunogenicity in therapeutic applications. Directed differentiation methods have made significant progress in recent years in the human side, allowing for the efficient and specific differentiation of iPSCs into a variety of cell types, including neurons (143), cardiomyocytes (144), and hematopoietic cells (145), among others. Therefore, direct differentiation of iPSCs represents a promising alternative for the development of new therapies and *in vitro* applications, holding the potential to significantly impact the field of regenerative medicine. However, directed iPSC differentiation presents several obstacles. To begin with, the process of inducing iPSCs to differentiate into specific cell types can be inefficient, with high variability, low specificity and poor reproducibility, as well as constituting a time-consuming and costly process (146). Furthermore, the differentiated cells may be heterogeneous and result in a population with varying degrees of differentiation and functional activity, and the presence of residual undifferentiated iPSCs can compromise the purity of the differentiated cell population (147). Overall, the lack of control over the differentiation process and the variability of iPSC lines pose significant challenges to their practical use.

To overcome the challenges in inducing differentiation of iPSCs, researchers are actively pursuing several strategies to improve the efficiency, specificity, and reproducibility of the process. The optimization of differentiation protocols is a crucial aspect of this research, as it involves refining the methods and conditions used to induce iPSC differentiation. This can involve adjusting the presence of specific growth factors that can influence the differentiation process or applying engineering-derived approaches to promote iPSC differentiation by mimicking the extracellular matrix (148). Directed differentiation is a strategy that involves directing iPSCs toward specific cell types using signaling pathways and small molecule inhibitors. This can help to increase the specificity of the differentiation process and reduce the formation of unwanted cell types, but requires deep knowledge on the embryonic development of the target cells to mimic the corresponding pathways (149), which is often complex and particularly in veterinary species. Reporter lines and cell sorting methods to identify and purify the cell population of interest is frequently used in human iPSC differentiation (150, 151). Including this approach into the strategies for veterinary iPSC differentiation would require further characterization of animal markers and species-specific antibodies to correctly identify the cells of interest. Another strategy is choosing iPSC lines that have a high propensity for differentiation into specific cell types, as has been described in the first part of the review (55). Finally, quality control measures are also essential for ensuring the purity of differentiated cell populations and minimizing contamination with residual undifferentiated iPSCs. This can be accomplished using molecular markers and other techniques that can help to distinguish between different cell types (119). Importantly, checking the identity of the obtained cells possesses its own challenges, as not all cell types exhibit a well-defined and stable pattern of markers, so several tests may be needed possibly including functional ones. Therefore, combining different strategies in a multi-faceted approach can help to address the challenges in the iPSC differentiation process from multiple angles, each one constituting a unique opportunity for research and development.

##### 3.2.1.1. iPSC-derived cells in companion animals

As commented above, different cell types have been derived from iPSCs in the equine and canine species, but not all of them have shown functional properties *in vitro* or *in vivo*. For instance, equine iPSCs have been differentiated *in vitro* into neurons, keratinocytes, myocytes, tenocytes, osteoblasts and chondrocytes. However, only neurons and myocytes have shown functional properties such as depolarization and contraction (128, 134). Equine tenocytes are apparently challenging to obtain (127) but mechanical loading can improve differentiation (63), and osteoblasts derivation can be promoted in 3D scaffolds that would also facilitate clinical application (129). Obtainment of chondrocytes from equine iPSCs has been limited and non-conclusive. Equine chondrocytes were obtained during spontaneous differentiation of iPSCs (60), but its derivation using an intermediate MSC stage has shown mixed results (60, 88, 152). More progress has been reported in the derivation of functional specific cells from canine iPSCs, including mature megakaryocytes able to release functional platelets (153).

Regarding all the considerations that have been discussed for iPSC differentiation, in companion animals it should be emphasized the need of further research into their embryological development to fine-tune differentiation protocols adapted to the particularities of each species, as well as on characterization of the obtained cells thus requiring species-specific reagents. Furthermore, the approach to generate iPSCs can later influence their differentiation potential, not only because of the epigenetic memory of the cell, but also because the permanent expression of transgenes may interfere with the differentiation process, which needs pluripotency silencing (154).

##### 3.2.1.2. Derivation of MSCs from iPSCs

Provided the complexity of deriving specific types of cells from iPSCs and the limitations of directly using undifferentiated iPSCs, an intermediate approach has been proposed: the derivation of MSCs from iPSCs, known as iMSCs. While this might look as a considerable round about, the use of iMSCs has several notable advantages over primary MSCs. In contrast to primary MSCs that are commonly obtained from more invasive sources like bone marrow or adipose tissue and require large quantities of tissue for isolation, iPSCs can be generated from small numbers of cells obtained from less invasive sources such as skin or blood. Furthermore, while primary MSCs are subjected to considerable variability among tissue sources, iMSCs can be derived from iPSC lines coming from single cell colonies, thus substantially increasing the homogeneity of the cell population and facilitating standardization of the cell product. In addition, primary MSCs do not possess a limitless self-renewal potential and enter senescence after some time expanding *ex vivo*. This requires collecting tissue again for MSC isolation and results in a new population of cells, even if the same donor is used. On the other hand, iMSCs can be derived from the same iPSC clonal line multiple times, and both iPSCs and iMSCs can be easily cryobanked for later use. In summary, iMSCs could be used for large scale production of homogeneous population of cells leading to phenotypical, molecular and biological stability that ultimately is needed for an ideal off-the-shelf product for therapeutic use (155, 156).

Despite of the advantages, the development of efficient and scalable methods for generating high-quality iMSCs remains a challenge in need of further investigation. Of note, MSCs found in adult tissues do not have all the same embryological origin. Most MSCs derive from the mesoderm, but some of them come from the paraxial mesoderm while others generate in the lateral plate mesoderm, or even in the extraembryonic mesoderm (157). Furthermore, the origin of some MSC populations has been traced back to neural crest cells generated in the ectoderm (158). These observations are important to understand the natural heterogeneity of MSCs, as well as to define strategies to derive them *in vitro* from iPSCs. General approaches for iMSC generation have been reviewed elsewhere (159) and are beyond the scope of this review, however it is worth highlighting that the derivation methodology can influence the properties of the resulting iMSCs. While main characterization features seem to be preserved, the functional properties of iMSCs may differ from their natural counterparts and among derivation strategies (160). This could constitute a limitation, but also an opportunity to direct iMSCs toward specific characteristics depending on the intended use.

Mesenchymal stromal cells from iPSCs have been generated in horses and dogs to obtain multipotent progenitor cells readily available for therapeutic use. Lepage et al. (152) used equine fetal fibroblasts to generate equine iPSCs lines that were subsequently differentiated into iMSCs. These displayed a typical fibroblast morphology, testing positive for CD29, CD44, and CD90 surface markers, and when tested for tri-lineage differentiation were able to differentiate into osteogenic and adipogenic lineage while failing to achieve chondrogenesis in 3D pellet culture (152). Similarly, Chung et al. (88) generated equine iMSCs by serial passaging of iPSCs in MSC-defined media which were characterized as CD29 and CD44 positive and were able to differentiate successfully into the chondrogeneic, osteogenic, and myogenic lineages (88). On the canine side, iMSCs have been generated by inhibiting the TGF-β/Activin pathway in MSC-defined media following serial passaging. Once generated, the canine iMSCs expressed CD73, CD105, STRO1+ and CD24 (135), with variable expression of CD90 and CD44, and did not express the pluripotency marker Oct3/4 as well as the negative surface markers CD45 and CD34 (89). All the canine iMSC lines generated in these studies showed tri-lineage differentiation *in vitro* and, interestingly, when canine iMSCs were compared with BM-MSCs, there was evidence of different time and concentration-dependent effect of dexamethasone and BMP-2 on the onset of osteogenesis, which needs to be taken into consideration for the generation of clinically relevant cells (90). The risk of uncontrolled *in vivo* differentiation would not be an issue since injected iMSCs locally in immunocompromised mice and systemically in healthy Beagle dogs did not form any teratomas or abnormal tissues showing potential for therapeutic safety (89). Although limited, the number of published studies shows the feasibility to generate successfully iMSCs in companion animals.

In addition to the interest on iMSCs for therapy, in human medicine patient-specific iMSCs have been exploited significantly as platforms for drug screening and toxicity for multiple conditions affecting mesenchymal lineages such as osteogenesis imperfecta (161), Fanconi anemia (162), fibrodysplasia ossificans progressiva (163), and Hutchinson-Gilford progeria syndrome (164). Based on this, future studies are needed in companion animals to assess efficiently the quality and optimize the large-scale production of iMSCs-based therapeutics and research platforms.

#### 3.2.2. Master banks of iPSCs for veterinary applications

Genetic mutations that cause the disease can be present in the starting cells, which could be transmitted to the newly generated iPSCs. While this is valuable for disease modeling applications by generating patient-specific iPSCs, it turns out to be a barrier for therapeutic use of autologous iPSCs. Furthermore, the quality of somatic cells to generate autologous iPSCs can be diminished by such genetic diseases or by aging, leading to a reduced yield of functional iPSCs and increasing the risk of rejection. This, in turn, can prolong treatment timelines and negatively affect therapeutic outcomes (165, 166). Moreover, the complexity and cost of generating iPSCs make it currently unpractical to produce these cells from the own patient, added to the prolonged time required to obtain, expand and characterize the iPSCs significantly delaying the treatment (167).

To overcome these limitations, allogeneic therapy has been proposed as a more feasible alternative. iPSCs can be generated from a healthy donor and then used to treat multiple patients. This eliminates the need for individualized cell sourcing, reducing the time and costs associated with autologous therapies. Additionally, allogeneic iPSCs can be characterized in detail prior to banking them to ensure their identity and quality, which otherwise would add significant further delay to the autologous treatment (167). Stem cell banks increase the availability and ensures the quality of the cell products, while reducing the time to administer the therapy (168). Not only iPSCs can be banked, but also their derivatives including iMSCs (169) and some types of differentiated cells (170), as well as their secretomes (171). However, it is important to note that allogeneic therapy also has its own limitations, highlighting the risk of immunological rejection (167). Even though the immune responses generated against allogeneic iPSCs and their derivatives requires further investigation, various strategies are being developed to overcome this potential hurdle. The use of immunosuppressive drugs and the genetic engineering of cells to reduce their immunogenic potential have been suggested. These approaches may be effective but also raise several concerns, such as drug side effects or further manipulation of the cell’s genome (172). Therefore, the focus could be put on the selection of donors.

Haplobanks have been proposed as a solution to provide a more widely available source of allogeneic iPSCs. The underlying idea is to select donors who are homozygous for the most common haplotypes for the MHC. While the genetic diversity of MHC haplotypes is high, some haplotypes are more common within a population. Identifying which haplotypes are more prevalent and banking iPSCs from healthy donors carrying such MHC types can allow providing MHC-matched cell products to a considerable part of the population (173, 174). This way, by using a limited number of selected donors, haplobanks can reduce the genetic diversity of the iPSCs and limit the risk of immunological rejection. Furthermore, the iPSCs stored in haplobanks can be differentiated into various cell types, which can then be used for transplantation or *in vitro* disease modeling (175). For instance, a clinical-grade iPSC haplobank in Japan has been established from seven donors and can provide HLA-matched iPSCs for approximately 40% of the Japanese population. This haplobank was released in 2015 and since then has provided iPSCs for over 10 clinical trials (176). This strategy could be transferred the veterinary field owed the growing knowledge on MHC haplotype diversity in different breeds of companion animals (177–180). To the best of our knowledge, iPSC haplobanks for companion animals are not yet a reality, but there are initiatives to create haplobanks for veterinary MSCs. The impact of MHC matching in MSC therapy in veterinary patients is being increasingly acknowledged (181–184), and the most common MHC haplotypes have been defined in several equine populations (177, 178). Following this path, the same concept could be implemented for animal iPSCs in the coming years.

#### 3.2.3. iPSC-based cell-free therapy

Another alternative to the limitations posed by iPSCs for their clinical application is the utilization of extracellular vesicles (EVs) or the entire secretome obtained from iPSCs, or from their derivative cells such as iMSCs or other cell types (185). It has been proposed that cells mainly communicate through their secretome, which consists of either packed or free components. The packed secretome, also known as EVs, are nano-sized sacs produced by a wide range of cell types, including different stem cells with therapeutic potential. EV-based therapy has gained substantial attention in recent years due to their benefits over traditional cell therapy, as it allows a cell-free modality that overcomes concerns related to immunogenicity and cell survival, and increases product standardization. The EVs comprise a diverse range of biologically active substances, including proteins, lipids, and nucleic acids, that can be delivered directly to target cells, leading to a desired therapeutic outcome (186). As intermediaries in cell therapy, EVs transmit information similar to their cells of origin. In comparison to cell therapy, where cells need to survive, migrate and differentiate for a therapeutic effect to occur, EVs can be more easily delivered to the site of injury because of their smaller size, either through direct injection or intravenous administration, without being recognized by the host immune cells. In the case of iPSC-based therapy, using their secretome also prevents additional concerns related to this specific type of cells, like their tumorigenicity (187).

Induced pluripotent stem cell-derived EVs offer the possibility of personalized medicine by producing patient-specific EVs and can be engineered to contain specific drugs or components. Despite this is a relatively new field needing further work, iPSC-derived EVs have been investigated in human regenerative medicine for various diseases, including osteoarthritis, skin and auto-immune disorders (188–190), however their application in companion animals has yet to be fully explored. To date, only one study has been conducted in dogs, serving as canine model for using human iPSC-derived cell-free secretome to enhance post-pneumonectomy compensatory response. Human iPSC-derived secretome showed improved angiogenesis and alveolar remodeling leading to enhanced gas exchange after the pneumonectomy (191). This highlights the therapeutic potential of iPSC-derived secretome and EVs in human and veterinary patients, particularly due to their ease of administration. Nevertheless, further research is necessary to fully realize their potential and to scale-up and standardize the production.

#### 3.2.4. Manufacturing of iPSCs for veterinary applications

The clinical adoption of robust and high quality iPSCs and their cell-derivatives requires a standardized and reliable cell Good Manufacturing Process (cGMP). Due to their ability to proliferate indefinitely, these cells can represent a true off-the-shelf product that can be manufactured in high number of identical doses from one cell line (192). To achieve this, the intrinsic challenges elaborated in details above for the generation of iPSCs need to be addressed in a large-scale-up context needed for cGMP. These aspects have not been assessed and published for companion animals, however, they have been implemented for human cGMP manufacturing and important considerations can be translated (112). For example, careful choice of the starting tissue source is fundamental and for large-scale manufacturing it would be ideal to use one less prone to chromosomal aberrations and epigenetic memory such as umbilical cord blood or peripheral blood due to sampling accessibility. The variability in cell reprogramming strategies represents a bottleneck and ideally, a process ensuring genomic and phenotypic stability would need to be implemented using non-integrating vectors or peptide-based delivery of transcription factors that can be easier to standardize for regulatory approvals. The laborious manual selection of iPSC colonies based on morphology would need to be automated in a high accuracy and robust process based potentially on micro-devices able to perform immunoselection for clinical grade sorting, and the *in vivo* teratoma assay could be replaced by a rapid qPCR throughput testing (193). The costs and standardization of cGMP manufacturing in general represent a limiting factor in veterinary medicine as also species-specific differences are a critical factor that needs to be taken into consideration. The differentiation toward an intermediate cell type such as iMSCs could be a beneficial step since guidelines for MSCs manufacturing in veterinary medicine have been published (102) and these can be easily translated into the process.

## 4. Discussion and conclusion

The application of iPSCs in the veterinary field is clearly less developed than in the human side. While this is not surprising because this is a complex and relatively new field, it seems that iPSC advancement in companion animals substantially faces bigger challenges. When thinking about the whole process of developing an application using iPSCs for companion animals, one can realize that there are additional hurdles since the very beginning. The generation of iPSCs in dogs, horses or cats has proved to be less effective than in humans. While transgene-free methodologies for reprogramming are already customarily used to obtain human iPSCs, in the veterinary side the integrating virus methods remain the most common. Furthermore, even for this approach, there are no standardized protocols yet and different methodologies with variable outcomes are reported, plus the same approach does not always work depending on the cell type (59). Even when putative iPSCs are generated, their characterization can show mixed results suggesting that some lines may not be fully reprogrammed, as it is also pointed out by the permanent transgene expression detected in some works (24). Hurdles continue after iPSC lines are established and ready for application. The pluripotent nature of these cells carries higher risks of tumorigenesis than the use of adult stem cells, and immunogenicity can be a concern not only in the allogeneic scenario but also in autologous application (26), which may compromise the safety of the therapy. Furthermore, xeno-free systems need to be further developed in veterinary iPSCs to reduce the risk of xeno-antigens.

Some of these limitations can be overcome by using cells already differentiated, derived from iPSCs. While theoretically iPSCs can be differentiated into any cell of the body, highly specific protocols are needed to provide the exactly required conditions, being the development of such protocols another field of great importance (194). A relatively easier alternative can be the derivation of iMSCs, which can greatly increase the availability, standardization and homogeneity of these cells compared to primary sources (160). Furthermore, manufacturing and cell banking strategies could be transferred from primary MSCs to iMSCs, or even to iPSCs and/or their derivatives like EVs (187). Creation of cell banks would facilitate the availability of different cells and cell products for several applications while reducing the time for treatment by using allogeneic cells. In this regard, the creation of haplo-banks to match donors and patients by their MHC has been proposed in human iPSCs (173) and is gaining consideration for veterinary MSCs, so this could also be transferred to veterinary iPSCs.

Considering the gaps in the field of iPSCs in companion animals, we propose five main areas in which focus needs to be placed:

First, we need to acknowledge and understand the differences between animal species and human. While extrapolating methodologies from the human side is common in veterinary research and certainly useful at initial stages, it is important to unveil the differences and work toward them. Better understanding on companion animal embryology is of utmost importance both to understand the pluripotency networks of ESCs and to direct the differentiation of iPSCs toward the desired cell lineage. Therefore, we need more basic research on this area and more work to transfer that knowledge into application in the iPSC generation, characterization and differentiation.

Second, more basic research is also needed to characterize clinically relevant cell types in companion animals. Most iPSC applications are based on deriving cells, either for therapy or for *in vitro* research like disease modeling. We need not only to understand how to derive these cells, but also to develop the tools to ensure their identity and, importantly, their functionality.

Third, even though it is crucial to firstly laying the foundations, it is equally important to start developing tools to keep building the field in the future. In this sense, parallel efforts are needed to establish approaches allowing implementation of iPSC applications in companion animals. Optimization and standardization of protocols for veterinary iPSC generation and characterization are very much needed, but in a later stage we will also need the tools to scale up the production of these cells and their derivatives in xeno-free conditions, using cGMP manufacturing and banking.

Fourth and closely related to all of the above, another area in need of improvement is the production and validation of species-specific reagents, like growth factors, antibodies, or other molecules needed during the processes of generation, characterization, expansion, and differentiation of iPSCs in companion animals. While there are some commercially available products suitable for veterinary species, the increasing specialization requires further development of custom solutions. This constitutes an interesting opportunity of collaboration with industry, and is an integral part of the cell therapy field in veterinary medicine.

Last, but not the least, promoting collaboration among researchers working in the veterinary iPSC field is key. Provided the many challenges we face, the best way to advance is to do it together by sharing expertise and resources, as well as experiences and failures. Some actions in this direction could include establishing a network of researchers and creating task forces, as well as considering the creation of bio-resources like cell banks, while seeking funding to support these actions. As in the fourth point above, the involvement of industry could also bring interesting opportunities to develop such networking and collaborations by taking advantage of industrial management skills and resources.

In conclusion, we are in front of a field of great promise that can significantly contribute to developing new therapies for veterinary patients, but also to providing critical information for human medicine. The veterinary field can greatly benefit from the advancements in the human side, but we also need to appreciate the differences and conduct basic research, while getting ready for the application.

## Author contributions

LB and FB contributed to the conception and structure of the manuscript. LB, TE, AO’B, and AI contributed to the literature revision and manuscript writing. TE designed the figures. LB, TE, AO’B, AI, and FB critically reviewed the manuscript. All authors contributed to the article and approved the submitted version.

## Funding

This project has received funding from the European Union’s Horizon 2020 research and innovation program under the Marie Sklodowska-Curie grant agreement no. 101026825.

## Conflict of interest

The authors declare that the research was conducted in the absence of any commercial or financial relationships that could be construed as a potential conflict of interest.

## Publisher’s note

All claims expressed in this article are solely those of the authors and do not necessarily represent those of their affiliated organizations, or those of the publisher, the editors and the reviewers. Any product that may be evaluated in this article, or claim that may be made by its manufacturer, is not guaranteed or endorsed by the publisher.
